# Zero-Contrast TAVR: Inching Toward Safer TAVR in Patients With Kidney Disease

**DOI:** 10.1016/j.jscai.2024.102251

**Published:** 2024-08-14

**Authors:** Nicholas Kassis, Marwan Saad

**Affiliations:** a Lifespan Cardiovascular Institute, Providence, Rhode Island; b Department of Medicine, Division of Cardiology, Warren Alpert Medical School of Brown University, Providence, Rhode Island

**Keywords:** chronic kidney diseases, renal failure, transcatheter aortic valve replacement

The expanding indications of transcatheter aortic valve replacement (TAVR) to treat aortic stenosis (AS) warrants continued improvement in patient safety and outcomes, particularly among those at disproportionally high risk. Patients with chronic kidney disease (CKD) are considered one such high-risk group that is well-represented among patients with severe AS. Although data have emerged demonstrating the reversibility to baseline CKD after TAVR,^1^ patients with impaired baseline renal function are at elevated risk of developing postoperative acute kidney injury, and the presence of both portend worse in-hospital,^2^ midterm,^3^ and long-term^1^ outcomes following TAVR. Albeit multifactorial in etiology, post-TAVR acute kidney injury has been linked to a modifiable risk factor, namely the perioperative exposure to nephrotoxic iodinated contrast from screening cardiac gated computed tomographic angiography (CTA) and intraoperative aortography. This has yielded novel methods to reduce or eliminate contrast use before and during TAVR. Several single-center pilot studies have demonstrated the safety and feasibility of zero-contrast TAVR.^4^, ^5^, ^6^, ^7^, ^8^, ^9^ As an alternative to contrast-enhanced CTA, these studies employed a multimodality imaging approach for annular sizing, which included some combination of contrast-free computed tomography (CT),^4^, ^5^, ^6^, ^7^ cardiac magnetic resonance (CMR),^4^^,^^7^^,^^8^ and intraoperative transesophageal echocardiography (TEE).^4^^,^^6^

In this issue of *JSCAI*, Elison et al^10^ approached zero-contrast TAVR by utilizing a preprocedural noncontrast CT chest to size the valve, intraprocedural pigtail catheter as a reference depth for valve deployment, and transthoracic echocardiography to evaluate postdeployment valve function. In a training cohort of 100 patients and a validation cohort of 60 patients with advanced CKD not yet on renal replacement therapy, the authors developed an algorithm that incorporated the sinotubular junction (STJ) and sinus of Valsalva diameters as well as sex to determine valve sizing in patients undergoing balloon-expandable SAPIEN valve implantation. The algorithm was then examined in a series of 35 patients at high risk of requiring hemodialysis who underwent TAVR implantation with moderate sedation and no contrast.

The results of the current study are promising. First, the model exhibited a strong correlation with actual valve size (training cohort R = 0.81 and validation cohort R = 0.76). Second, in a rather sick population (mean age 79 ± 11 years, mean eGFR 21.5 ± 9.2 mL/min/1.73 m^2^, 31.2% with reduced left ventricular systolic function, 14.3% with concomitant cardiogenic shock, and 57.1% undergoing urgent/emergent TAVR), favorable clinical outcomes were achieved, including 100% technical procedural success rate and no intraoperative mortality, valve embolization, annular rupture, need for second valve, significant paravalvular leak, or access site complications. Four (11.4%) patients developed complete heart block requiring permanent pacemaker placement, and 1 (2.9%) patient experienced a stroke. Importantly, only 2 (5.7%) patients required new hemodialysis post-TAVR. It is critical to note that those favorable outcomes were achieved without the complementary utilization of CMR, TEE, or balloon valvuloplasty for valve sizing.

The question is, can this algorithm be universally used for patients with severe AS at risk of renal replacement therapy undergoing TAVR? Unfortunately, the study has several limitations that make answering with a simple “yes” rather unrealistic. First, the study was conducted at a high-volume center, and the algorithm was designed only for balloon-expandable SAPIEN valves in a small cohort of patients with tricuspid AS. As the authors noted, this limits the generalizability of the protocol to other centers, valve platforms, and patients with bicuspid AS or prior surgical aortic valve replacement (valve-in-valve TAVR).

Second, the proposed valve sizing algorithm had an average risk of 9.4% undersizing and 14.4% oversizing; hence, the precision and safety of the algorithm should be interpreted with caution, particularly as oversizing is known to be associated with an increased risk of annular rupture,^6^^,^^11^ coronary obstruction,^12^ and conduction disturbances requiring permanent pacemaker implantation.^13^ A recent feasibility study that similarly employed noncontrast CT to size self-expanding valves among 25 patients who underwent transfemoral TAVR reported an oversizing rate of 36%,^4^ and to exclude annular rupture and coronary occlusion, multiple safety checkpoints with contrast CT were implemented, and contrast aortogram was utilized to confirm valve positioning.^4^ In this vein, it is evident that relying solely on noncontrast CT for TAVR planning and implantation may not be sufficient to minimize the associated risks, and multimodality imaging should be considered when feasible. As stated by the authors, prior studies demonstrated that CMR provides a near-perfect correlation between annular sizing and resultant valve size selection compared with contrast CTA, and in the absence of head-to-head studies comparing varying imaging modalities, CMR appears to be the safer contrast-free study of choice. If CMR is not readily feasible, such as in the sick patient cohort of the current study, concomitant preoperative or intraoperative TEE may be considered to confirm annular sizing.

Lastly, a significant limitation of noncontrast CTA is its inability to precisely evaluate the risk of coronary obstruction with TAVR. Important risk indicators, such as coronary heights, valve-to-coronary distance, valve-to-STJ distance, and leaflet-STJ mismatch,^14^ will be nearly impossible to measure in the absence of contrast CTA. Moreover, relying on imperfect surrogates such as clinical symptoms, electrocardiographic changes, or regional wall motion abnormalities on transthoracic echocardiography may not be sufficient to ensure the absence of coronary obstruction after valve deployment. Intraoperative TEE could serve well in confirming patent coronary ostia when there is a concern.

The investigators should be commended for their efforts in advancing the field by offering a novel method to enhance the safety and applicability of zero-contrast TAVR in patients with underlying renal dysfunction. This promising algorithm should be regarded as a proof-of-concept rather than practice-changing; however, it represents an important step for larger multicenter studies to perform head-to-head comparisons between various isolated and combined imaging modalities to determine the safest and most effective approach to performing TAVR in this high-risk patient population. Ultimately, we will likely find that, as with most things in medicine, the approach should be personalized—in this case, to the patient’s risk profile, valve type, and institutional expertise.
